# Construction of the first high-density genetic linkage map of *Salvia miltiorrhiza* using specific length amplified fragment (SLAF) sequencing

**DOI:** 10.1038/srep24070

**Published:** 2016-04-04

**Authors:** Tian Liu, Linlin Guo, Yuling Pan, Qi Zhao, Jianhua Wang, Zhenqiao Song

**Affiliations:** 1 State Key Laboratory of Crop Biology, Shandong Key Laboratory of Crop Biology, Shandong Agricultural University, Taian, Shandong, China; 2 Agronomy College, Shandong Agricultural University, Taian, Shandong, China

## Abstract

*Salvia miltiorrhiza* is an important medicinal crop in traditional Chinese medicine (TCM). Knowledge of its genetic foundation is limited because sufficient molecular markers have not been developed, and therefore a high-density genetic linkage map is incomplete. Specific length amplified fragment sequencing (SLAF-seq) is a recently developed high-throughput strategy for large-scale SNP (Single Nucleotide Polymorphisms) discovery and genotyping based on next generation sequencing (NGS). In this study, genomic DNA extracted from two parents and their 96 F1 individuals was subjected to high-throughput sequencing and SLAF library construction. A total of 155.96 Mb of data containing 155,958,181 pair-end reads were obtained after preprocessing. The average coverage of each SLAF marker was 83.43-fold for the parents compared with 10.36-fold for the F_1_ offspring. The final linkage map consists of 5,164 SLAFs in 8 linkage groups (LGs) and spans 1,516.43 cM, with an average distance of 0.29 cM between adjacent markers. The results will not only provide a platform for mapping quantitative trait loci but also offer a critical new tool for *S. miltiorrhiza* biotechnology and comparative genomics as well as a valuable reference for TCM studies.

*Salvia miltiorrhiza* Bunge, also known as Danshen in Chinese, is a typical herb plant that is an important traditional Chinese medicine (TCM). This plant has been used extensively for thousands of years to treat various diseases: particularly coronary heart disease and cerebrovascular diseases in China and Japan, and recently in the United States, and many European countries^1^. Currently, more than 114 compounds have been isolated, of which diterpenoid quinones and hydrophilic phenolic acids are the major constituents^2^. Moreover, recent studies have found several new bioactivities of Danshen constituents, such as antioxidant, antitumor, and protective effects on the kidney and liver, suggesting the potential for new applications^3^. More than 320 Danshen preparations are produced by different pharmaceutical manufacturers.

There is constantly increasing demand for *S. miltiorrhiza* because of its varied and diverse pharmacologic properties. Currently, the annual demand for Danshen in China is approximately 10 million kg. Interest in biotechnology research on Danshen is increasing in many research groups. A large number of genes involved in the biosynthesis of phenolics and terpenoids have been identified through either molecular cloning or transcriptome-wide analysis^2^,^4^,^5^,^6^,^7^,^8^,^9^,^10^,^11^,^12^. However, the genetic background and regulators of these two biosynthetic pathways, especially in the later steps, remain unknown. Danshen is of considerable research interest, and its superior genetic characteristics, such as its modest genome size, low number of chromosomes, vitality, short generation cycle and mature tissue culture technology, have resulted in Danshen becoming a valuable plant among TCM^13^. Some researchers regard *S. miltiorrhiza* as an ideal model plant for TCM classes and labiatae plants^13^.

At present, almost all of the Danshen preparations that are consumed are primarily obtained by extraction from plants. An important and urgent task is to focus on improvements in Danshen for optimizing desirable traits, e.g., effective components, resistance and yield.

According to the literature on *S. miltiorrhiza*, several molecular markers, including amplified fragment length polymorphisms (AFLPs), sequence-related amplified polymorphisms (SRAPs), EST-simple sequence repeats (SSRs) and inter-SSRs (ISSRs), have been used to analyze the genetic diversity of Danshen. Previous studies have shown that *S. miltiorrhiza* is a cross-pollinated plant with high differentiation of its germplasm^14^,^15^,^16^. These results provide an important basis for further construction of genetic maps with the aim of selecting parents and markers.

Genetic linkage maps, particularly high-density genetic maps, are one of the most valuable tools in meeting the requirement of high-throughput superior trait selection among various germplasms, including plants and animals. The genetic map of Danshen, even though it is a model medicinal plant, was only recently constructed in our laboratory, and has just begun to be studied^17^. This map, which was constructed using SRAPs, ISSRs, and EST-SSRs in the F_1_ population, includes 94 loci with an average interval distance of 4.3 cM. However, this unsaturated genetic map has limited future application. Previous studies have demonstrated that an increased marker density can significantly improve the resolution of a genetic map in a given mapping population^18^. Additionally, the development of high-throughput sequencing technology provides the capacity for developing massive single nucleotide polymorphism (SNP) markers^19^.

SNPs are the most abundant and stable form of genetic variation in most genomes and have become the marker type of choice in many genetic studies^20^. Recently, specific length amplified fragment sequencing (SLAF-seq), a high-resolution strategy, was developed for large-scale *de novo* SNP discovery^21^. This approach has been successfully applied to high-density genetic map construction for many plants and animals regardless of the reference genome sequence. A high-density kiwifruit (*Actinidia chinensis*) SLAF-seq map has been constructed with 4,301 (SNP) markers^22^. A total of 8,007 SLAF markers were linked in the genetic map of mei (*Prunus mume*)^23^. Using 5,308 SLAF-seq prior markers, an ultra-high-density genetic map was developed for soybean by Qi *et al.*^24^. Zhang *et al.*^25^ successfully used SLAF-seq markers to construct a high-density genetic map for sesame. SLAF sequencing has also been successfully applied in other plants, including rice^26^ and cucumber^20^, even in animals such as chicken^27^ and shrimp^28^. This approach was also used to detect the QTL for the isoflavone content of soybean^29^. These results show that SLAF sequencing is a powerful high-throughput technique for the efficient development of a large number of polymorphic markers in a short time and is effective for linkage map construction.

In this study, SLAF-seq was used for the rapid discovery of SNPs in the F1 population. Subsequently, a high-density genetic map of *S. miltiorrhiza* was constructed, which contained 5,164 high-quality SLAFs and spanned 1516.43 cM, with an average marker interval of 0.29 cM. The characteristics of this genetic map are analyzed and discussed in detail in this study. The methods used in this study for genetic mapping and for the development of markers provide a valuable reference for other medicinal plants.

## Results

### Analysis of SLAF-seq data and SLAF markers

The control sequencing data were evaluated to ensure the validity of the SLAF library construction. HaeIII and Hpy166II were used for the SLAF library construction, according to the results of the SLAF pilot experiment. For the control in this study, the ratio of paired-end mapping reads was 76.25%, the percentage of digestion was 93.50% and the ratio of reads in the prediction range was 71.53%. The construction of the SLAF library was robust.

A total of 155.96 Mb of data containing 155,958,181 pair-end reads with a length of 100 bp were generated for *S. miltiorrhiza.* The Q30 ratio was 89%, and the GC (guanine-cytosine) content was 41.96%. The number of reads in the male and female parents was 11,105,419 and 9,795,546, respectively. On average, 1,406,846 reads were generated in the F1 mapping population. In the male parent, the number of SLAFs was 112,166, and the average depth of each SLAF marker was 39.15-fold. In the female, 110,726 SLAFs were generated, with an average depth of 32.73-fold for each SLAF. An analysis of the F1 mapping population indicated that 83,154 SLAFs were generated, with an average depth of 6.17-fold for each offspring (Table 1).

**Figure 1:**
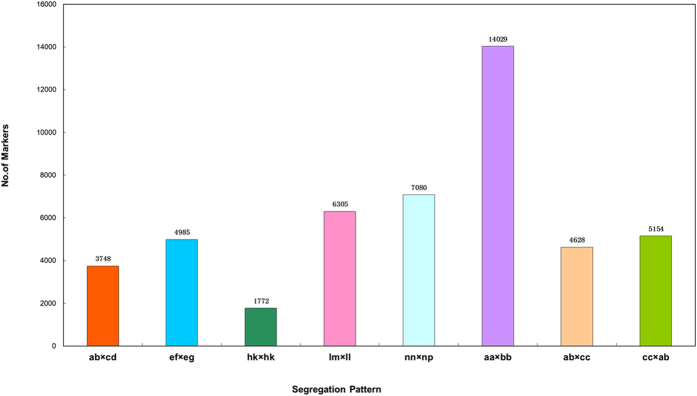
Numbers of markers for eight segregation types. The x-axes indicate the segregation types and the y-axis indicates the number of markers.

Among the 151,035 high-quality SLAFs, 62,834 were polymorphic, resulting in a polymorphism rate of 41.60%. Of the 62,834 polymorphic SLAFs, 47,701 were classified into eight segregation patterns (Fig. 1). For the F1 population, five segregation patterns (ab × cd, ef × eg, hk × hk, lm × ll, nn × np) were used for genetic map construction, and 23,890 SLAFs fell into these classes (a ratio of 15.82%). The genotyping quality scores (see **Materials and Methods** for details) were used to select the qualified markers. A total of 5,198 SLAFs were used for map construction (Table 2, Supplementary Table S1).

### Basic characteristics of the genetic maps

After completing the data preparation, 5,164 of the 5,198 SLAFs were mapped onto the genetic map (i.e., a ratio of 99.34%). There was a total of 2,966 BH18 (male) markers, 3,038 ZH74 (female) markers, and 5,164 SLAFs (7,554 SNPs), which fell into 8 LGs, for the integrated map. The coverage of the markers was 75.67-fold in the female parent, 91.20-fold in the male parent and 10.36-fold in each F1 individual (on average). The final map was 1,516.43 cM in length, with an average inter-marker distance of 0.29 cM (Figure S1).

The map contained 8 LGs that were equal to the gamete chromosome number of Danshen, but differed in length, this unequal length is identical to the uneven length of the karyotypic parameters of Danshen chromosomes^14^. The largest LG, LG8, harbored 830 markers covering a length of 297.25 cM; the largest average inter-marker distance was 0.36 cM. LG1 was the most saturated, comprising 559 markers with an average marker density of 0.24 cM, and the smallest LG, LG5, contained 478 markers with a length of 130.64 cM and an average inter-marker distance of 0.27 cM. The largest LG in the parental map was the same as that of the integrated map, LG8, with 468 markers covering 297.244 cM for the male and 511 markers covering 293.022 cM for the female. However, the smallest LG varied widely, corresponding to LG1 for BH18 (107.028 cM) and LG7 for ZH74 (115.325 cM) (Table 3).

Based on the map length methods of Postlethwait *et al.*^30^ and Chakravarti *et al.* (1991), the total expected length was 1519.044 cM. The cover rate of the map length was 99.83%. Comparing our previous report of *S. miltiorrhiza* genetic map, which is located 93 markers covered 400.1 cM with an average distance of 4.3 cM per markers, the density for this linkage map is very high. Especially, there is a largest gap, i.e., 20.67, was measured in LG8 between Marker 45299 and Marker 24265 with a high recombination rate (12.5%), which corresponds to recombination hotspots for this population.

### Evaluation of the genetic map

To evaluate the quality of the genetic map, haplotype mapping and heat mapping were performed. A haplotype map reflects the population with double crossovers, which are caused by genotyping errors, suggesting a possible recombination hotspot. A total of 5,164 SLAF markers were used for the haplotype map construction, as described by West *et al.*^31^. The recombination events and missing events for each individual were also displayed in the haplotype maps. The double crossover and deletion rates were less than 0.02% for each linkage group (Table 4). The recombination relationship between markers from a single linkage group was reflected in the heat map using pair-wise recombination values for the 5,164 mapped SLAF markers, which identified ordering errors.

## Discussion

### Characteristics of SLAF-seq for large-scale marker development

A genetic map provides an important foundation for quantitative trait locus (QTL) mapping, but the utility of genetic linkage maps relies primarily on the types and numbers of polymorphic markers used. Several conventional molecular markers, such as AFLPs, SRAPs, ISSRs, and EST-SSRs, exhibited high efficiency for analyzing *S. miltiorrhiza* genetic diversity and were then used to construct a genetic map^17^. The limited quantity of available markers renders the construction of a high-density genetic linkage map for *S. miltiorrhiza* almost impossible using conventional methods. With rapid development in sequencing technology, high-density polymorphic single nucleotide polymorphism (SNP) markers are now being used in several species for large-scale genotyping and high-density genetic map construction^20^.

The SLAF-seq strategy, a combination of locus-specific amplification and high-throughput sequencing, has been subjected to a series of critical trials to guarantee its high efficiency, accuracy and density^21^. Based on a careful analysis of the genomic GC content, repeat conditions and genome length of *S. miltiorrhiza.*, RsaI and HaeIII were selected to digest the genomic DNA with a digestion rate of 93.50%. Subsequently, SLAFs (264–334 bp) were selected in a pilot experiment for further paired-end sequencing; these SLAFs represent 71.53% of all SLAFs. A pre-designed scheme and a pilot experiment were conducted to ensure the density, uniformity and efficiency of the marker development. According to a minimal sequencing depth of 6-fold for each individual in the SLAF-seq strategy^21^, the sequencing depth of the parents and progenies in our study exceeded 83-fold and 10-fold, respectively. In addition, the average genotype quality score of all SLAF markers reached the cut-off value of 30, which was sufficient to filter the reads with low sequencing depth. Thus, the combination of sequence depth and genotype quality scores sufficiently enhanced the genotyping accuracy. Using high-throughput SLAF sequencing, we developed 151,035 high-quality SLAFs, of which 62,834 were polymorphic. A total of 5,198 polymorphic SLAFs were identified for linkage map construction.

Our results clearly demonstrate SLAF-seq as an acceptable tool for large-scale genotyping and for the rapid development of a large number of efficient markers, thus meeting the requirements for genetic map construction. Therefore, the present study extends the utility of SLAF sequencing to medicinal plant species and will be of interest to others working with herb plants.

### Value of the high-density genetic map of *S. miltiorrhiza*

*S. miltiorrhiza* is a TCM model and an economically important medicinal crop^32^. Thus, the research on *S. miltiorrhiza* basically represents the overall level of current Chinese medicinal research. Only one genetic linkage map has been published thus far, and this map has a low saturation level^17^. Several studies have shown that HighMap can be successfully applied to high-density linkage mapping using SLAF markers^20^,^23^,^25^.

Segregation distortion indicates that the genotypic frequency deviates from a typical Mendelian ratio. Segregation distortion is a common phenomenon that has been observed in many studies. Markers with segregation distortion frequently affect the accuracy if they are used to construct genetic maps because this deviation may be caused by gametic selection and/or zygotic selection^33^,^34^. Due to a limited quantity of available molecular markers and/or a low polymorphism rate, markers with segregation distortion may be included in the construction of high-density genetic maps under the precondition of unchanged original linkage groups^20^,^25^. Numerous studies have shown that markers with segregation distortion help to improve the detection of linked QTLs. In this study, markers with significant segregation distortion (P < 0.01) were initially excluded from the map construction, therefore guaranteeing maximum map accuracy.

In this study, a high-density genetic map was developed using the SLAF-seq method for genotyping. Compared with the previous map containing 93 markers^17^, the number of mapped loci (93 vs. 5164), the marker density (4.3 cM vs. 0.29 cM), and the total map length (400.1 cM vs. 1516.43 cM) were significantly improved in the SLAF genetic map. In addition, according to the formula for genetic linkage maps^30^, the coverage ratio of the total length to the expected length was 99.83%, which is considerably stronger compared to the value of 84.4% obtained for the first map^17^. The current linkage map covers nearly the entire genome with a resolution of 0.29 cM. To our knowledge, this is the first high marker-density map of *S. miltiorrhiza*. Our results showed the SLAF-seq strategy was showed a powerful method for marker discovery and high-density linkage map development. What’s more, the results not only provide numerous markers for *S. miltiorrhiza* but also data for QTL mapping and molecular breeding of specific agronomically important traits.

Moreover, the genome of Danshen has been completely sequenced, and the genome length is estimated to be 630 Mb. However, a physical map has not been reported. The high resolution of this map and the high-throughput sequences with long lengths may also provide a valuable reference for the construction of a fine-scale physical map through the positioning of sequence scaffolds and assistance in the assembly process of the genome sequence^20^.

Similar to the low genetic research level of the TCM model, the vast majority of TCM plants display generally weak genetic backgrounds, and no genetic maps have been reported. To our knowledge, genetic linkage maps have been established for only a few TCM species^35^,^36^,^37^,^38^,^39^, which were primarily derived using traditional molecular markers. We used SNP-seq to construct a high-density map for TCM; therefore, the present study provides a model or reference for the construction of genetic maps for medicinal plant species.

### Map strategy and mapping populations of medicinal plants

An apposite mapping population, which generally includes RIL, DH, F2 or backcrossed progeny, is very important in the construction of genetic maps^40^,^41^. However, similar to most medicinal species, it is very difficult to obtain a typical family-based population in *S. miltiorrhiza* due to its high heterozygosity resulting from a long history of natural cross-pollination and inbreeding depression.

The double pseudo-testcross strategy was first proposed by Grattapaglia and Sederoff (1994) and was successfully applied to construct a genetic map of forest trees. In the pseudo-test cross, an F1 progeny is developed as a mapping population by hybridizing two unrelated and highly heterozygous individuals, where gene segregation patterns can be interpreted as a backcross. This strategy has been widely used in plant species that lack appropriate pedigrees^41^,^42^,^43^,^44^,^45^,^46^,^47^.

In the present study, an F_1_ interspecific hybrid population of the above-mentioned *S. miltiorrhiza* lines with different characteristics was created, and 96 seedlings of the F1 family were used for SNP genotyping and for the construction of genetic linkage maps using the double pseudo-testcross mapping strategy. In the pseudo-testcross, the polymorphic markers fell into eight segregation patterns, five of which, i.e., ab × cd, ef × eg, hk × hk, lm × ll, nn × np, could be used for genetic mapping^44^,^45^. In our study, the rate of the five types was 50.08%. However, the most common segregation pattern (aa × bb), which was different from that observed in a traditional population such as F2, could not be used for mapping purposes.

Due to the limited number of available segregation patterns, a considerable difference between the parents was required to generate large polymorphic SLAFs. In the present study, we selected the lines ZH74 and BH18 because both agricultural trait and molecular genetic differences are highly obvious. A total of 330 amplified polymorphic primer pairs were detected among 550 primers, with a polymorphism rate of 46%^17^. In our study, among the 151,035 high-quality SLAFs, 62,834 were polymorphic, with a polymorphic rate of 41.60%; this result also reflects the considerable difference between the two lines. Compared with other plants, such as cucumber, with a polymorphic rate of 9.57%^20^, sesame, with 5.11% polymorphic SLAFs^25^, and mei (*Prunus mume*), with 40.35% polymorphic SLAFs^23^, the two lines used as mapping parents showed considerable differences, demonstrating that they met the requirements of mapping populations for further high-density map construction.

High diversity is primarily determined by high heterozygosity with natural cross-pollination^14^. Many medicinal plants have a cross-pollinating habit. There is no doubt that this tendency provides favorable conditions for the selection of mapping parents. If there is a poor genetic basis and the lack of a typical family-based population, the pseudo-testcross is the most promising method for creating genetic maps of medicinal plants at the present time.

## Conclusions

In this study, 151,035 high-quality SLAFs were developed using the SLAF-seq method for genotyping. Of these SLAFs, 62,834 were polymorphic. To our knowledge, we have constructed the first high-density genetic map for *S. miltiorrhiza* using an F_1_ population, which consisted of 5,164 markers in 8 linkage groups spanning 1516.43 cM. According to the analysis of the SLAFs and their sequence information, we conclude that SLAF-seq is an effective strategy for large-scale genotyping. Furthermore, this high-density genetic map will provide a foundation for further research on the fine mapping of genes/QTLs and molecular breeding. Importantly, the mapping population and the SLAF-seq application in our research provide valuable references for other TCM plants.

## Materials and Methods

### Plant material and DNA extraction

An F1 population consisting of 98 individuals was derived from an intra-specific cross of *S. miltiorrhiza* ‘BH18’ (male parent) and *S. miltiorrhiza f. alba* ‘ZH74’ (female parent) (Additional File 4). The ZH74 female plants are characterized by purple flowers, slender leaves, a relatively high root weight and low fat-soluble ingredients. The male parent (BH18; *S. miltiorrhiza bge. f. alba*) has white flowers, leaves with circular blades, a relatively high tanshinone content and a low root weight. In the spring of 2014, seedlings of the progeny and parents were planted in an experimental field of Shandong Agriculture University in Tai’an (N36.16, E117.16), Shandong Province, China, under standard conditions. Young healthy leaves from the two parents and F1 individuals were collected, stored in liquid nitrogen and then transferred to a freezer at −70 °C until DNA extraction. Young leaves (0.5 g) from each plant were ground in liquid nitrogen, and DNA isolation and purification were performed using the CTAB (cetyl trimethyl ammonium bromide) method^48^. The yield and quality of DNA were estimated using an ND-1000 spectrophotometer (NanoDrop, Wilmington, DE, USA) and electrophoresis in 0.8% agarose gels.

### SLAF library construction and high-throughput sequencing

An improved SLAF-seq strategy was used in our experiment. First, the reference genome (http://www.ncbi.nlm.nih.gov/genome/?term=Arabidopsis%20thaliana) was obtained using software^21^ to ensure the yield, quality and uniformity of the SLAFs. Then, the SLAF library was constructed using a predesigned scheme. Two enzymes, RsaI and HaeIII (New England Biolabs, NEB, USA), were used to digest the genomic DNA of the two parents and the F1 population. Subsequently, a single nucleotide (A) overhang was added to the digested fragments using a Klenow Fragment (3′ → 5′ exo–) with dATP at 37 °C. Dual-index sequencing adapters (PAGE-purified, Life Technologies, USA) were then ligated to the A-tailed fragments using T4 DNA ligase. Polymerase chain reactions were carried out in reaction solutions containing the diluted restriction/ligation samples, dNTP, High-Fidelity DNA polymerase (NEB), and PCR primers 5′-AATGATACGGCGACCACCGA-3′ (forward primer) and 5′-CAAGCAGAAGACGGCATACG-3′ (reverse primer) (PAGE-purified, Life Technologies). Then, the PCR products were purified using Agencourt AMPure XP beads and pooled. The pooled samples, which were electrophoresed on 2% agarose gel, and SLAFs of 400–450 bp (including adapter sequence indexes and adaptors) were selected for paired-end sequencing on an Illumina HiSeq 2500 sequencing platform (Illumina, San Diego, CA, USA). Real-time monitoring was performed for each cycle during sequencing, and the ratio of raw high-quality reads with quality scores greater than Q30 (a quality score of 30, which indicates a 0.1% chance of obtaining an error and thus 99.9% confidence) and the guanine-cytosine (GC) content were calculated for quality control.

### Sequence data grouping and genotyping

SLAF-seq data grouping and genotyping were performed following Sun *et al.*^21^. All high-quality SLAF pair-end reads (quality score >30e) with clear index information were clustered based on sequence similarity detected using BLAT2 (-tileSize = 10 -stepSize = 5). Sequences with more than 95% identity were grouped into one SLAF locus as described by Sun *et al.* The single nucleotide polymorphism (SNP) loci of each SLAF were then detected between the parents, and SLAFs with more than 3 SNPs were filtered out first. Alleles were defined in each SLAF using minor allele frequency (MAF) evaluation. Because Salvia miltiorrhiza is a diploid species and one locus can only contain at most four SLAF tags, only groups with fewer than 4 seed tags were identified as high-quality SLAFs. In this study, SLAFs with a sequence depth less than 200 were defined as low-depth SLAFs and were filtered out in the following analysis. SLAFs with 2, 3, or 4 tags were identified as polymorphic SLAFs and were considered to be potential markers. Polymorphic markers were classified into eight segregation patterns (ab × cd, ef × eg, hk × hk, lm × ll, nn × np, aa × bb, ab × cc and cc × ab). Because our population for mapping was an F1 population obtained by a cross between two heterozygote parents, five segregation patterns (excluding aa × bb, ab × cc and cc × ab) were selected for genetic map construction.

To evaluate the genotyping quality, a Bayesian approach was proposed for the genotype score^21^. First, we calculated a posteriori conditional probability based on the number of single nucleotide polymorphisms and the coverage of each allele. Next, qualified markers for subsequent analysis were selected according to the genotyping quality score, which was based on the probability. Low-quality markers for each individual and each marker were counted, and the worst individuals and markers were deleted during the dynamic process. The process was terminated when the average genotype quality scores of all SLAF markers reached the cut-off value. Three strict criteria were used to filter high-quality SLAF markers for the genetic mapping. First, average sequence depths <10-fold in the parents were filtered out. Second, the integrity of the markers had to be >70%. Finally, markers with significant segregation distortion (P < 0.01) were initially excluded for the map construction.

### Linkage map construction

For the selected high-quality markers, the modified logarithm of odds (MLOD) scores <5 were filtered. Because the NGS data inevitably suffered from genotyping errors, a newly developed HighMap strategy was used to ensure a high-density and high-quality genetic map, which would correct the SLAF markers and order genotyping errors within the LGs^49^. Briefly, a two-point analysis was used to calculate the recombinant frequencies and LOD scores, which were applied to infer linkage phases; this process was then combined with spatial sampling, simulated annealing algorithms and enhanced Gibbs sampling to conduct an iterative process of marker ordering^50^,^51^. The mapping algorithm was terminated when all markers were appropriately mapped. According to the parental contribution of the genotypes, the error correction strategy of SMOOTH was then conducted^52^. To impute a missing genotype, a k-nearest neighboralgorithm was applied^53^. Skewed markers were then added to this map by applying a multipoint method of maximum likelihood^53^.

The markers that were heterozygous in both parents were regarded as anchor markers. The markers that were heterozygous in the female or male parent were used to construct sex-specific maps. Then, by integrating the parental maps through the anchor markers, a consensus map was established^51^. The Kosambi mapping function was used to estimate the map distances. The map distances for the anchor markers were calculated as the average of the two parental distances. Other markers were placed on the consensus map using interpolation or extrapolation according to the relative position between the flanking anchor markers on the relevant parental map.

### Availability of supporting data

Detail information of markers is included as supplementary information.

## Additional Information

**How to cite this article**: Tian, L. *et al.* Construction of the first high-density genetic linkage map of *Salvia miltiorrhiza* using specific length amplified fragment (SLAF) sequencing. *Sci. Rep.* **6**, 24070; doi: 10.1038/srep24070 (2016).

## Supplementary Material

Supplementary Dataset 1

Supplementary Information

## Figures and Tables

**Table 1:** SLAF-seq data summary for *Salvia miltiorrhiza.*

	Male parent	Female parent	Offspring
Total reads			
No. of reads	11,105,419	9,795,546	1,406,846
Reads in high-quality	4,390,756	3,624,211	512,867
Reads in repeat SLAFs	1,164,662	1,068,021	142,393
Reads in low depth SLAFs	324,888	358,746	58,482
High-quality SLAFs			
No. of SLAFs	112166	117026	83155
Average SLAF depth	39.15	32.73	6.17
Polymorphic SLAFs			
No. of polymorphic SLAFs	57333	57669	40515
Average depth in parent	33.61		
Average depth in individuals	5.93		
High-quality SLAF markers			
No. of high-quality SLAF markers	5164	5164	4950

**Table 2:** Numbers of each marker segregation type on the linkage map of.

Type	SLAF Number	Percentage(%)
ab × cd	75	1.44
ef × eg	425	8.18
hk × hk	341	6.56
lm × ll	2,143	41.23
nn × np	2,214	42.59
Total	5,198	100

**Table 3:** Genetic map for 19 linkage groups (LGs).

	Total Marker			Total Distance(cM)			Max Gap		
	Female (ZH74)	Male (BH18)	Integrated map	Female (ZH74)	Male (BH18)	Integrated map	Female (ZH74)	Male (BH18)	Integrated map
LG1	347	316	559	141.153	107.028	132.85	15.79	6.68	7.88
LG2	341	371	613	186.544	180.181	190.39	17.24	13.01	7.88
LG3	426	430	747	195.187	216.418	213.66	21.86	9.12	9.6
LG4	378	390	652	183.249	175.127	181.17	10.38	18.74	9.11
LG5	279	266	478	153.085	108.194	130.64	13.01	26.95	10.45
LG6	404	372	675	196.998	202.857	207.41	114	18.44	11.53
LG7	352	353	610	115.325	170.161	163.048	6.68	20.27	19.22
LG8	511	468	830	293.022	297.244	297.25	26.95	17.24	20.67
Total	3038	2966	5164	1,464.563	1,457.21	1,516.43	26.95	26.95	20.67

**Table 4:** Double crossover and deletion for 8 LGs.

Linkage Group ID	Singleton Percent(%)	Missing Percent(%)
LG1	0.01	0.01
LG2	0.01	0.01
LG3	0.00	0.01
LG4	0.00	0.01
LG5	0.02	0.00
LG6	0.00	0.01
LG7	0.00	0.01
LG8	0.00	0.00
