# Supplementary material for: Construction of the first high-density genetic linkage map of Salvia miltiorrhiza using specific length amplified fragment (SLAF) sequencing
Source: Sci Rep. 2016 Apr 4;6:24070. doi: 10.1038/srep24070 (PMC4819198; doi:10.1038/srep24070)
Supplement: Supplementary Information [file srep24070-s2.pdf]

Construction of the first high-density genetic linkage map of *Salvia miltiorrhiza* using specific length amplified fragment (SLAF) sequencing

LIU TIAN[1], GUO LINLIN[1], PAN YULING[1], ZHAO QI[1], SONG ZHENQIAO [1, 2]*, WANG JIANHUA[1, 2] *

Figure S1 Distribution of SLAF markers on eight linkage groups of *S. miltirrohiza*. The integrated map is between two male and female maps. Also, the genotypes are noted.

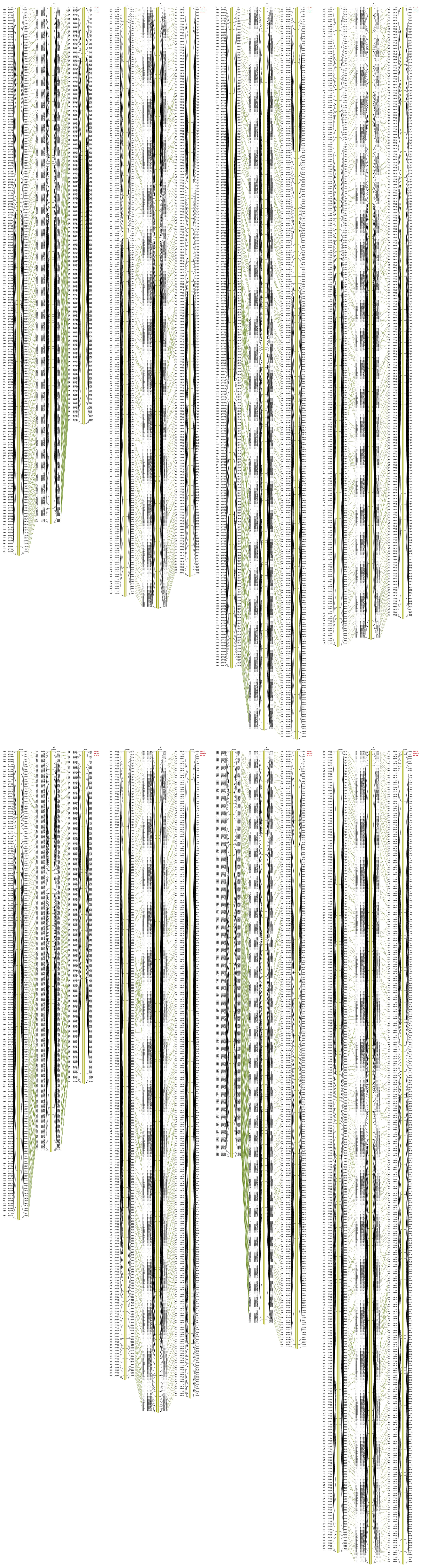

Addition file S2: Haplotype map of the genetic map.

Green represents BH18, blue represents ZH74, and white indicates that the parent could not be estimated, gray represents deletions, and red indicates heterozygosity.

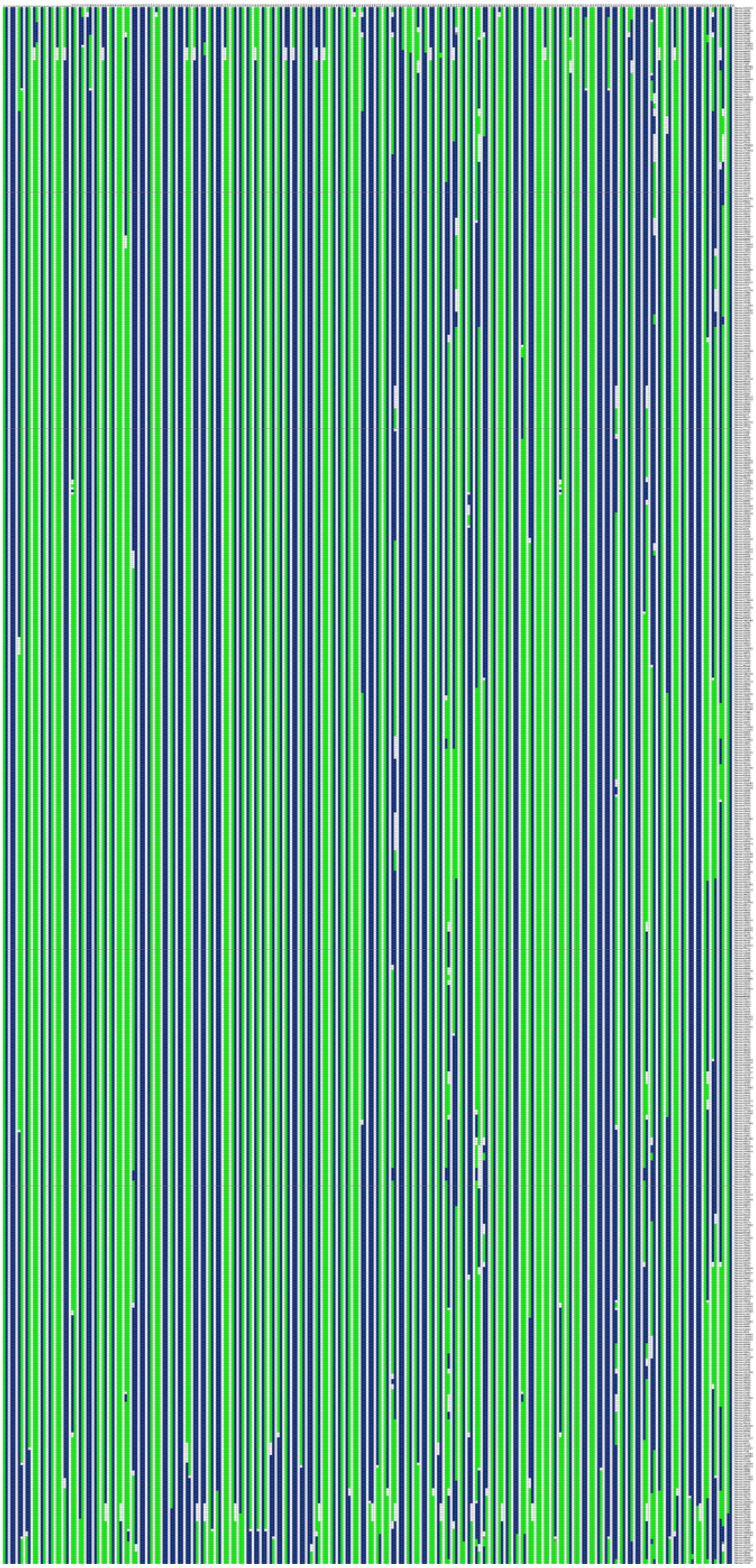

Haplotype map of the integrated map (LG2)

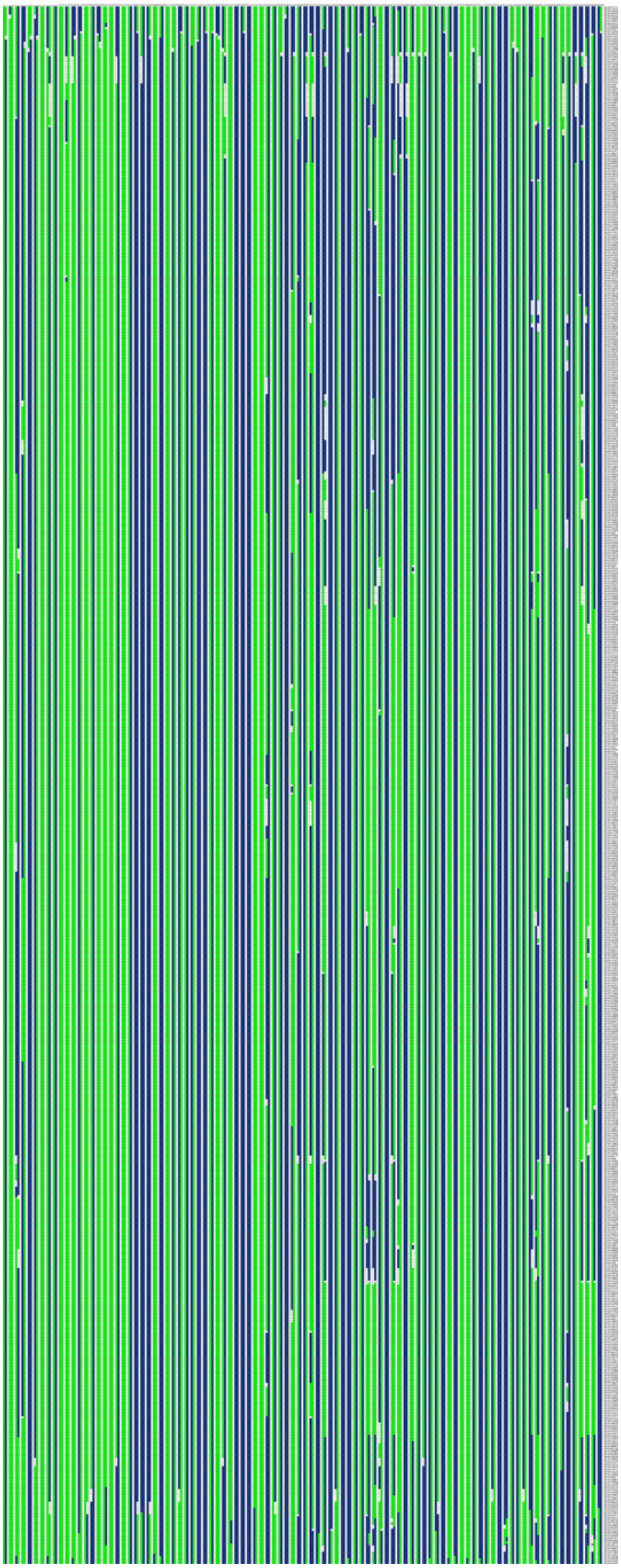

Haplotype map of the integrated map (LG3)

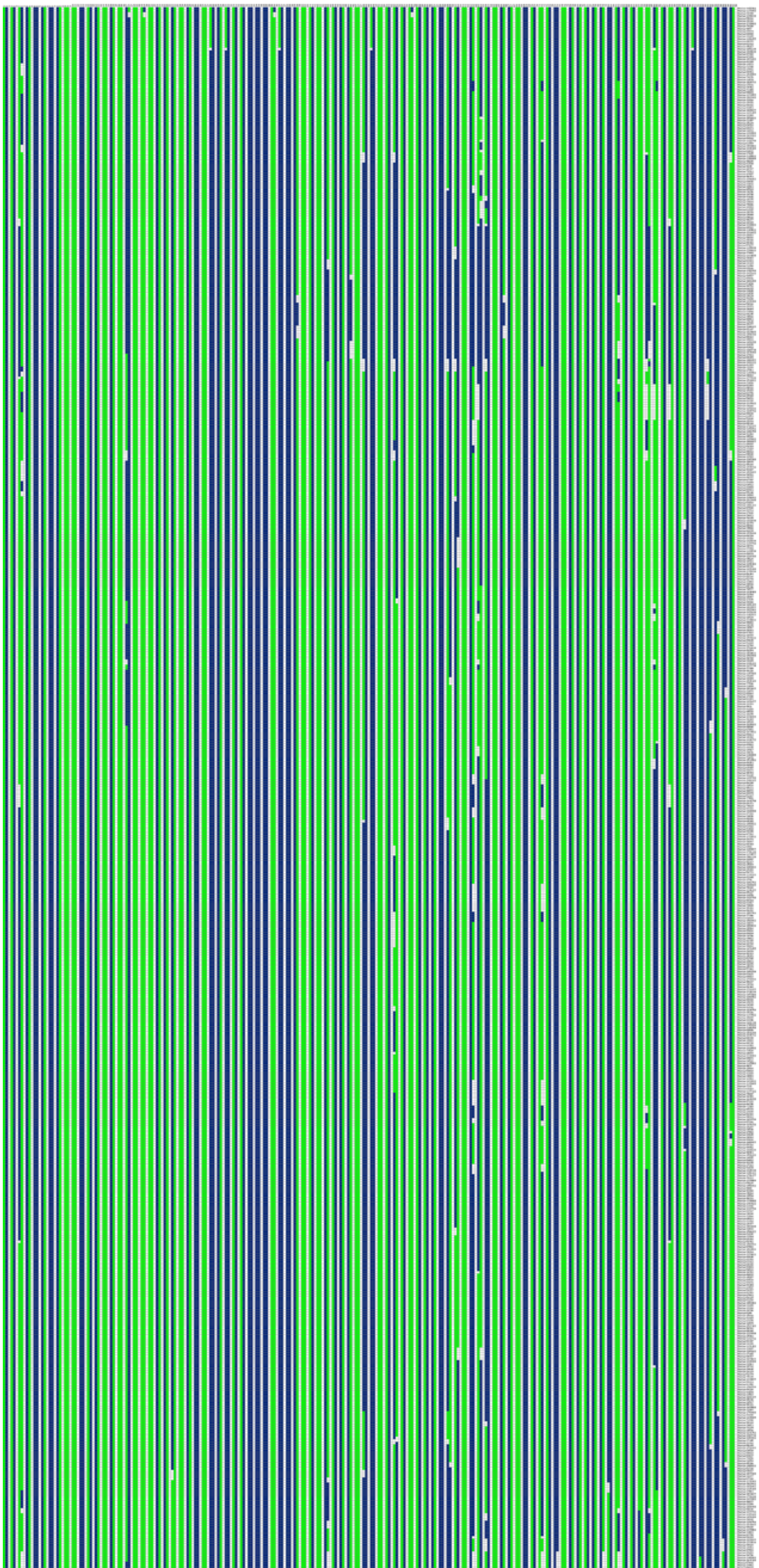

Haplotype map of the integrated map (LG4)

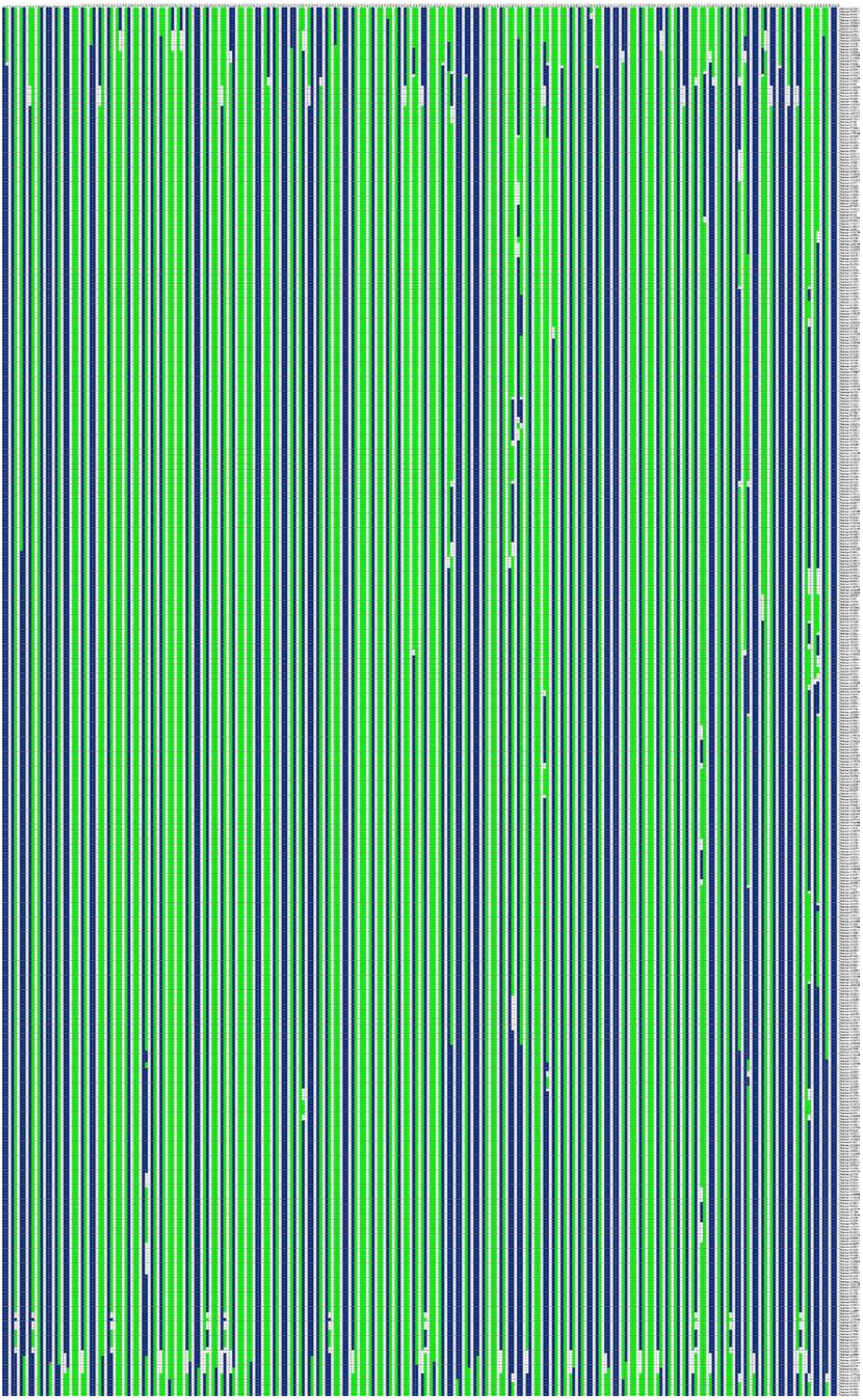

Haplotype map of the integrated map (LG5)

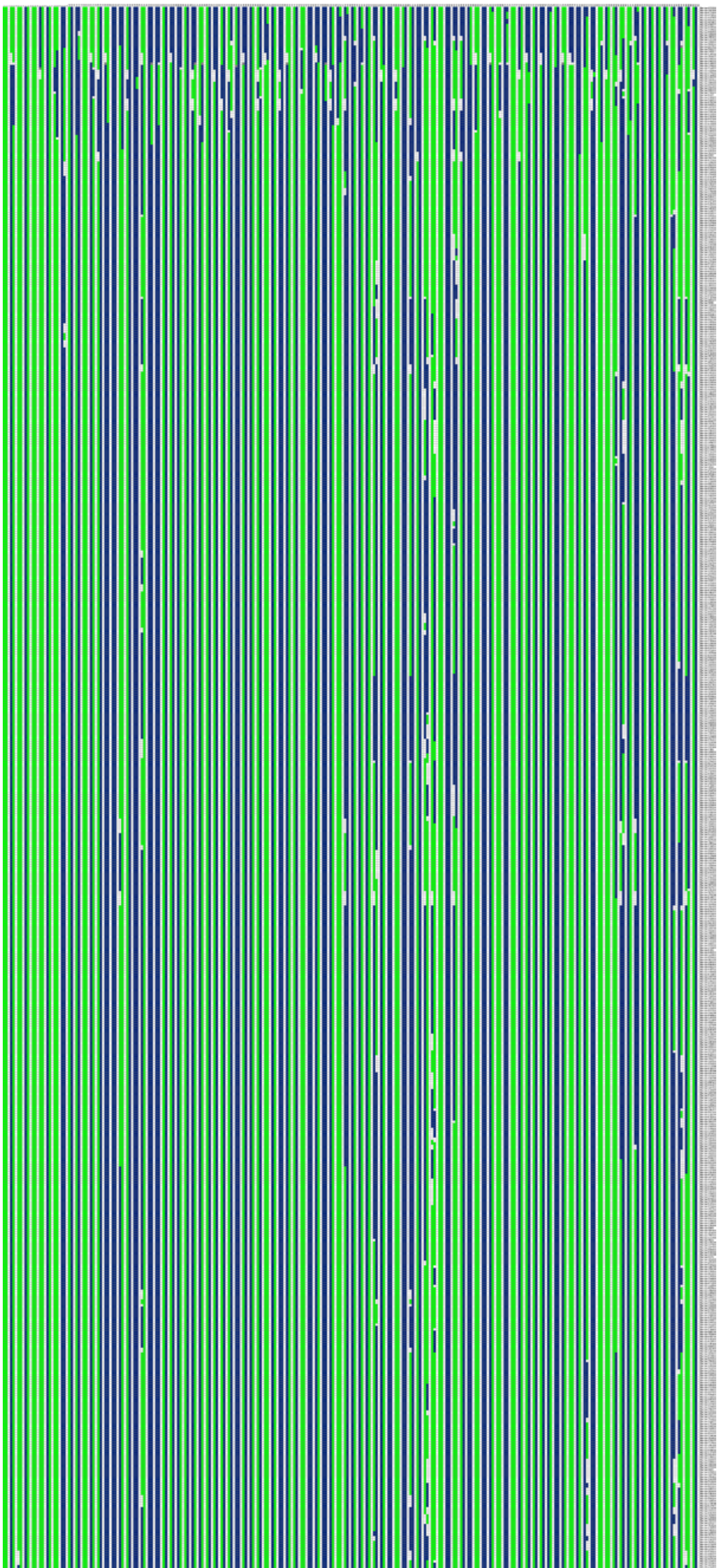

Haplotype map of the integrated map (LG6)

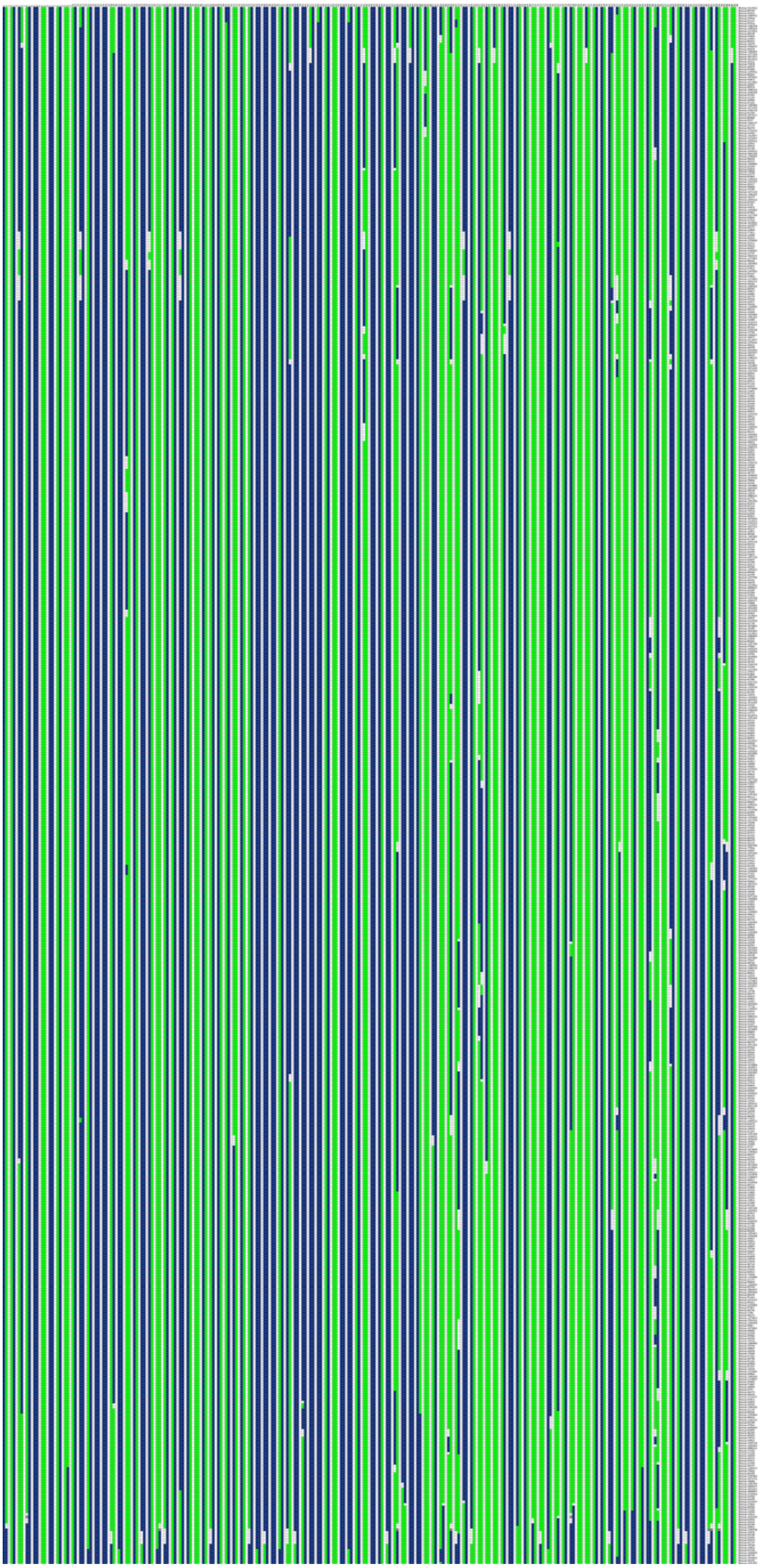

Haplotype map of the integrated map (LG7)

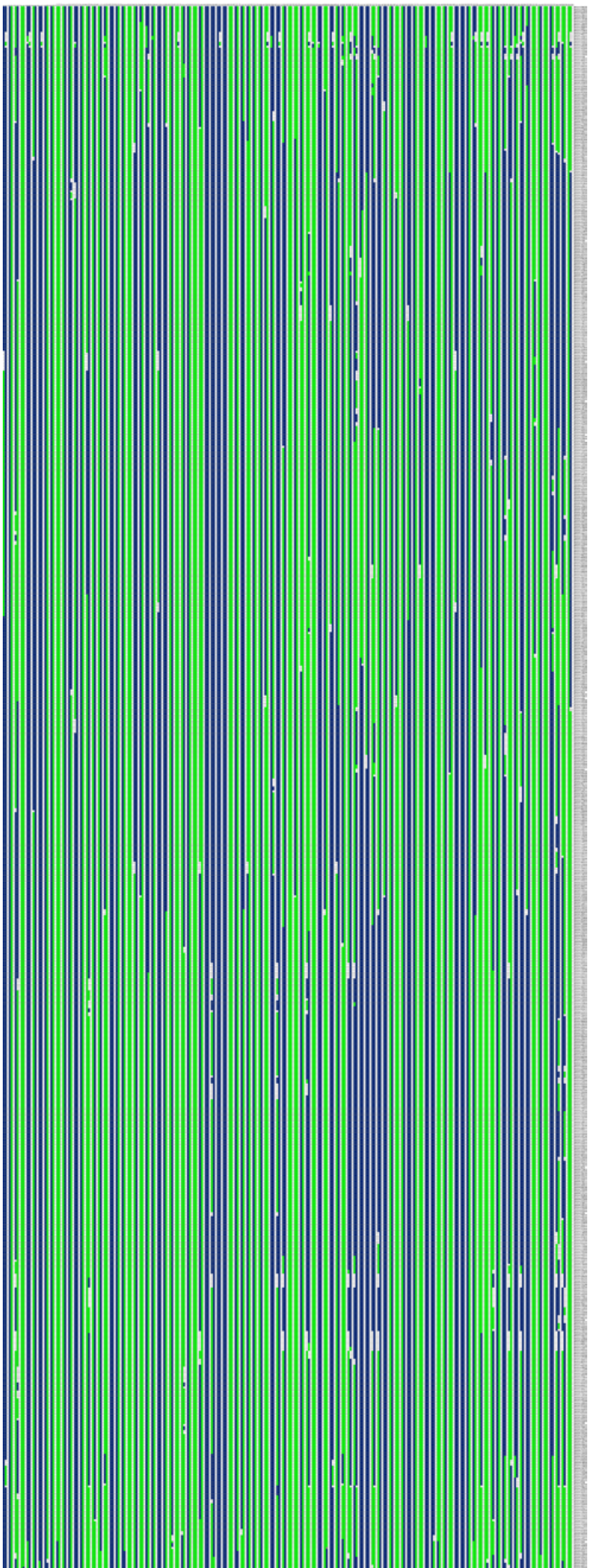

Haplotype map of the integrated map (LG8)

Addition file S3: Heat map of the genetic map.

Each cell represents the recombination rate of two markers. Yellow indicates a relatively low and purple indicates a relatively high recombination rate.

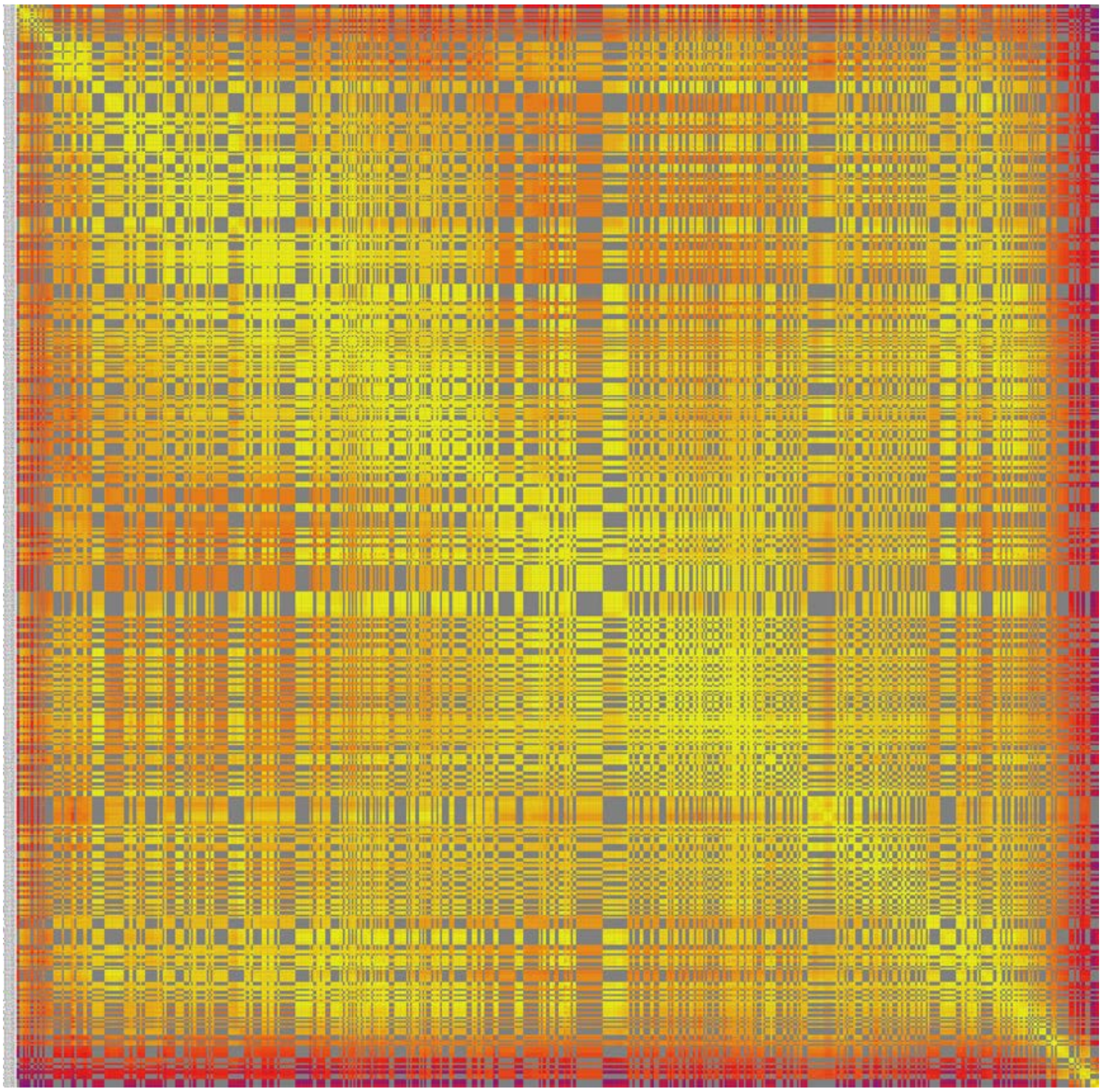

Heat map of the integrated map (LG2)

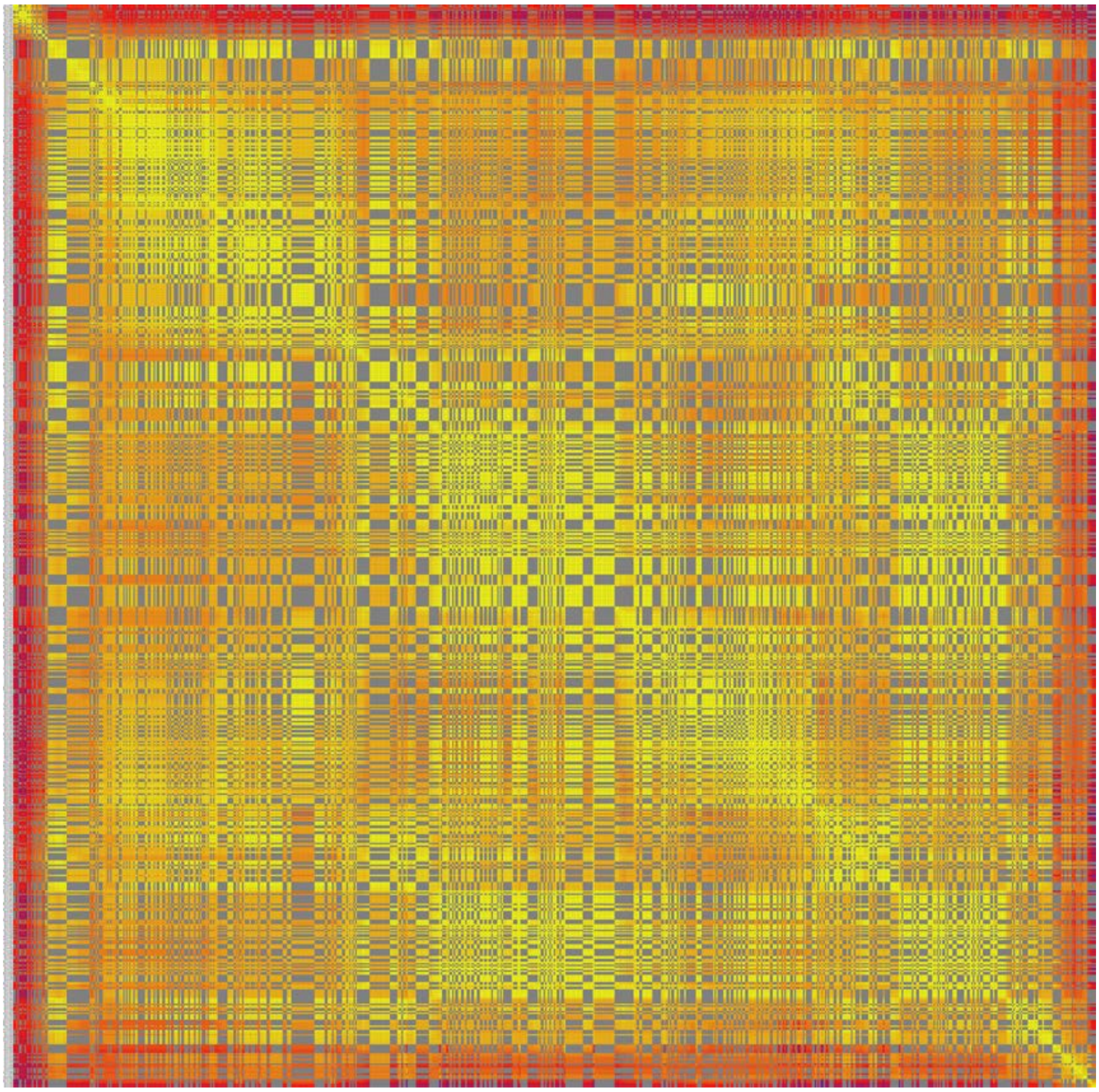

Heat map of the integrated map (LG3)

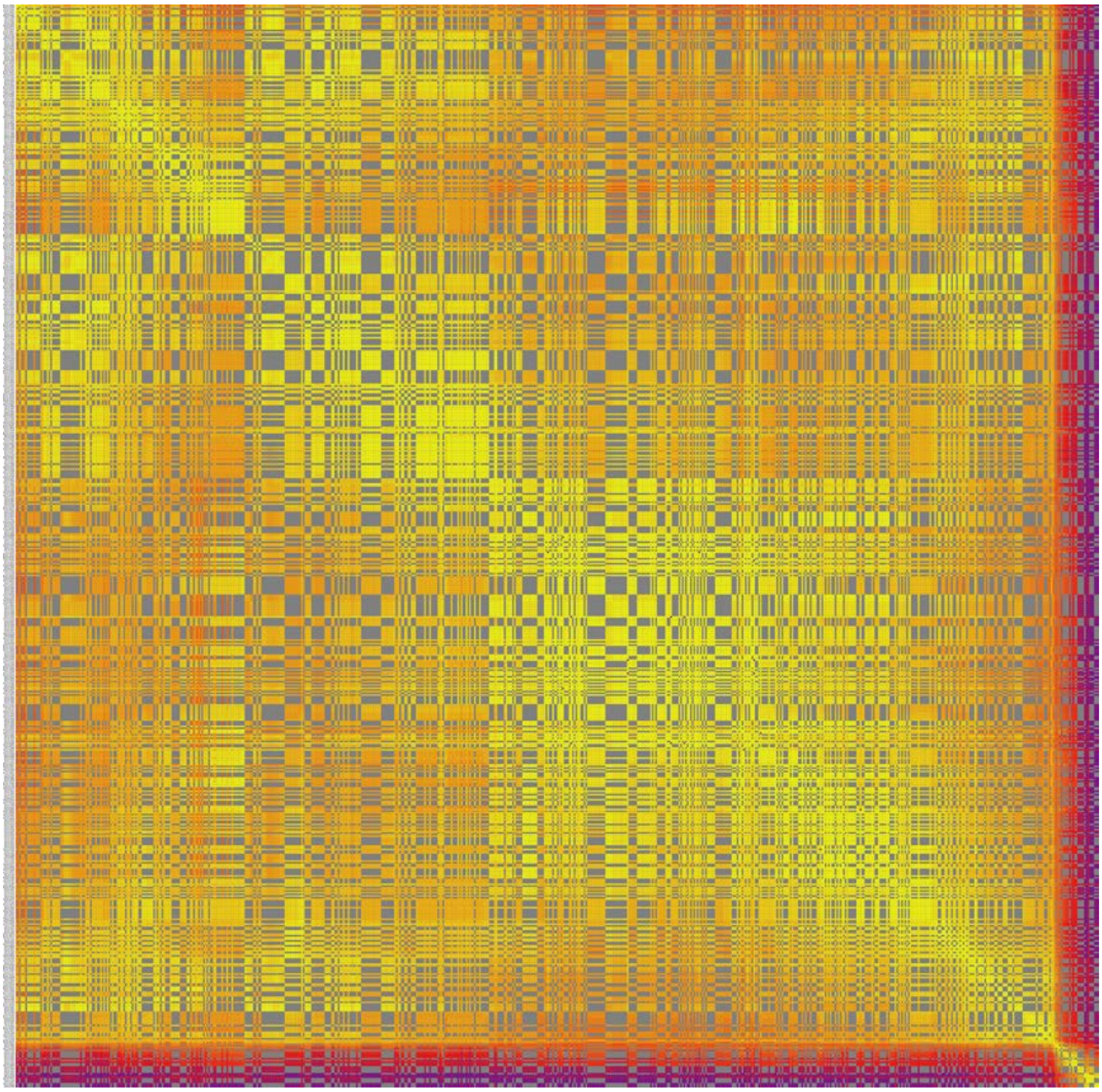

Heat map of the integrated map (LG4)

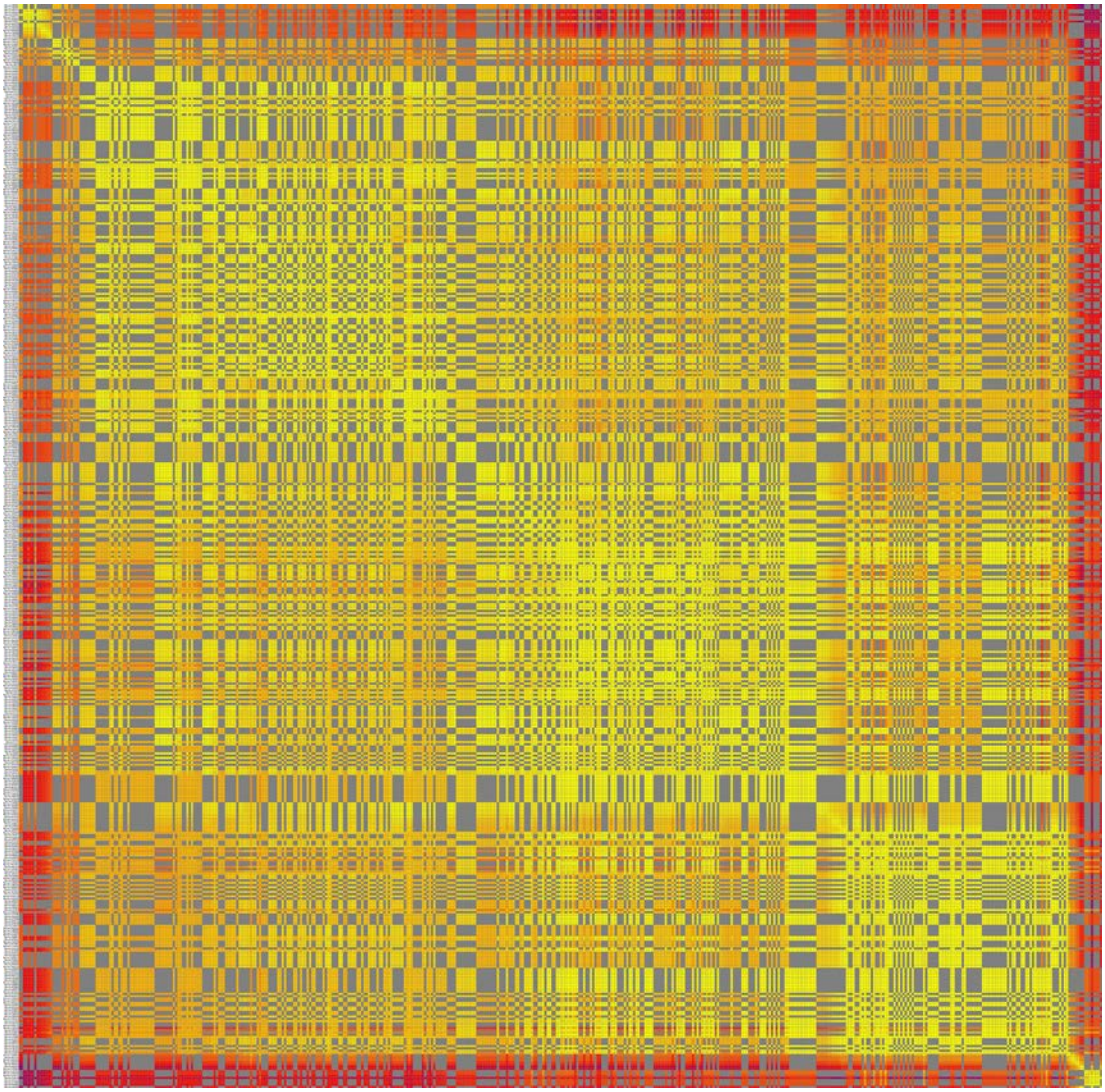

Heat map of the integrated map (LG5)

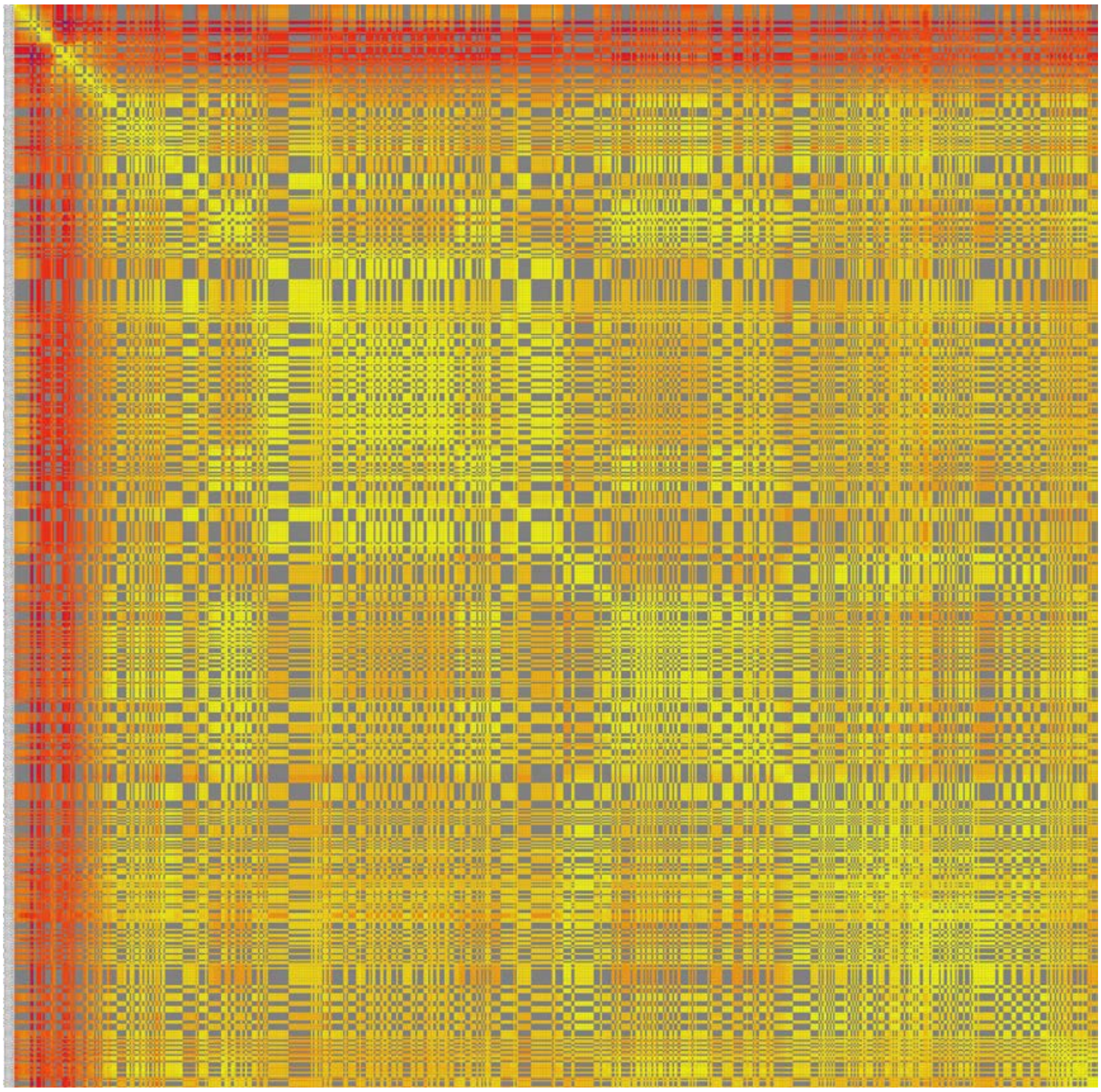

Heat map of the integrated map (LG6)

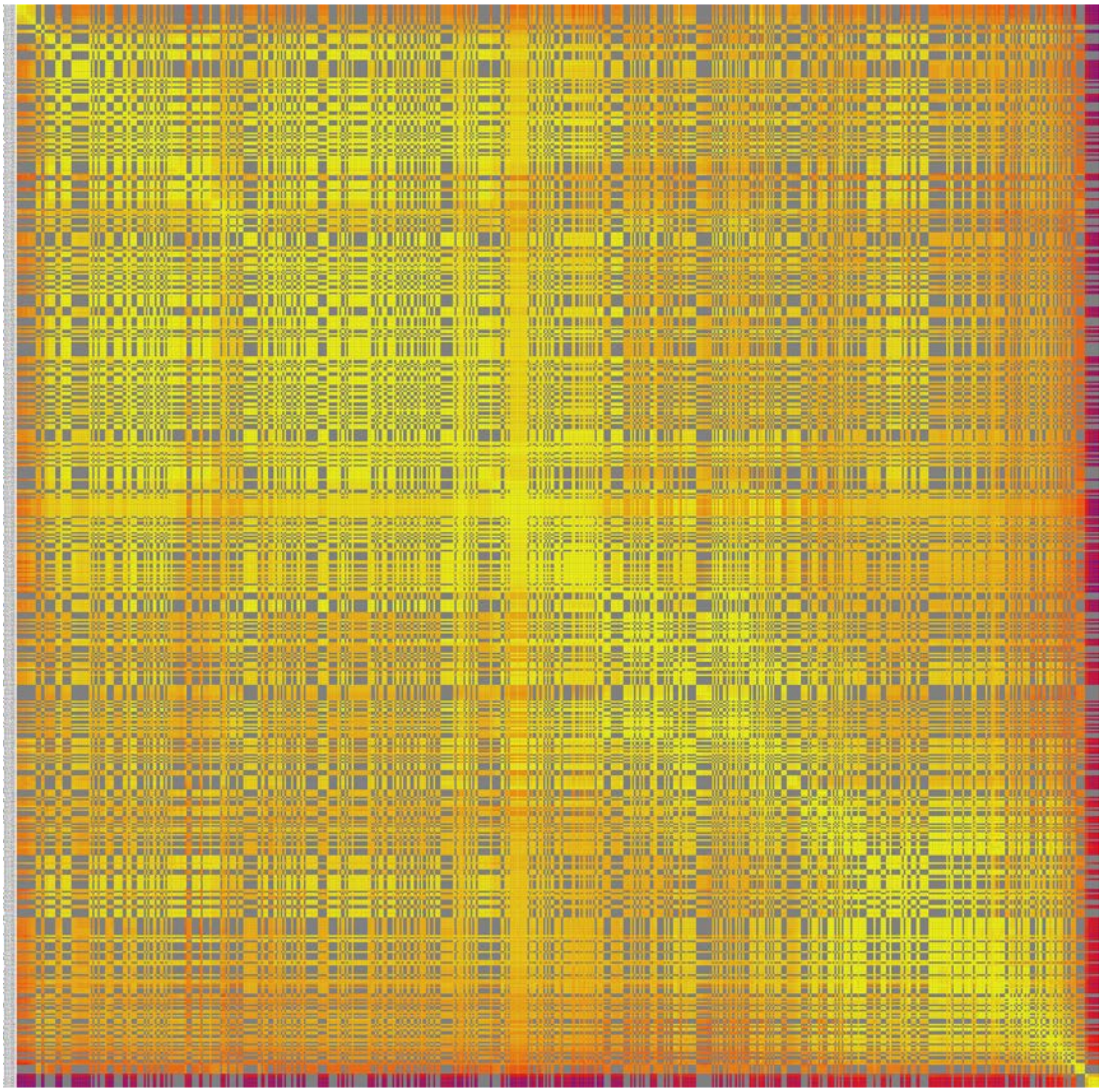

Heat map of the integrated map (LG7)

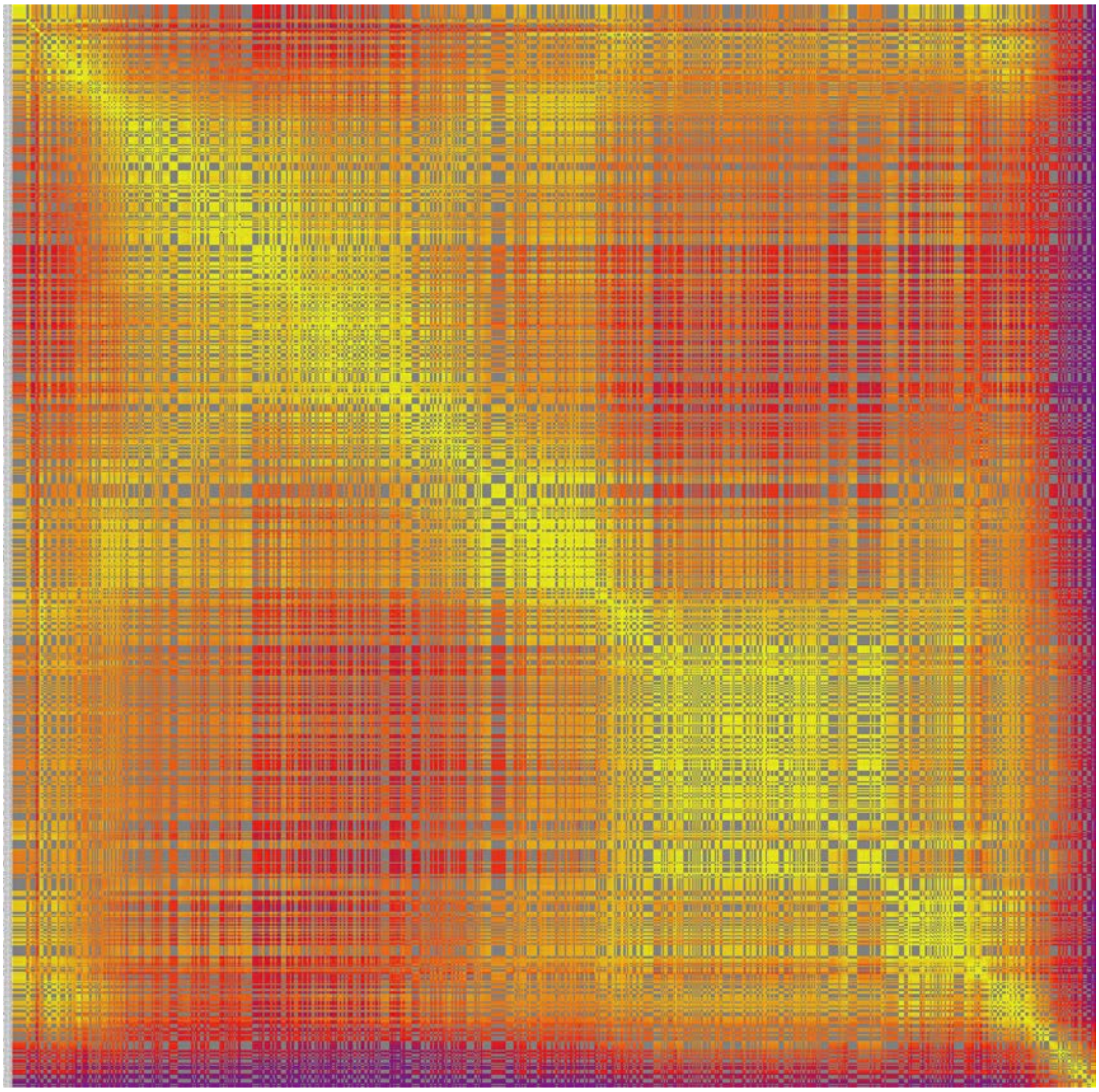

Heat map of the integrated map (LG8)

Addition file S4: Leaves and plant phenotype of the BH18, ZH74 and some $F_{1.}$

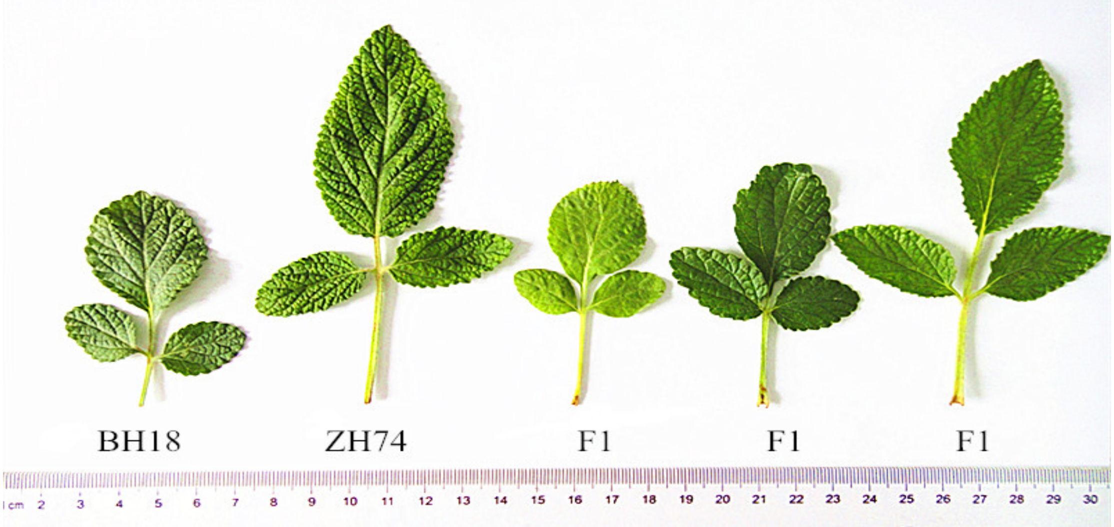

Leaves of BH18, ZH74 and some $F_{1.}$

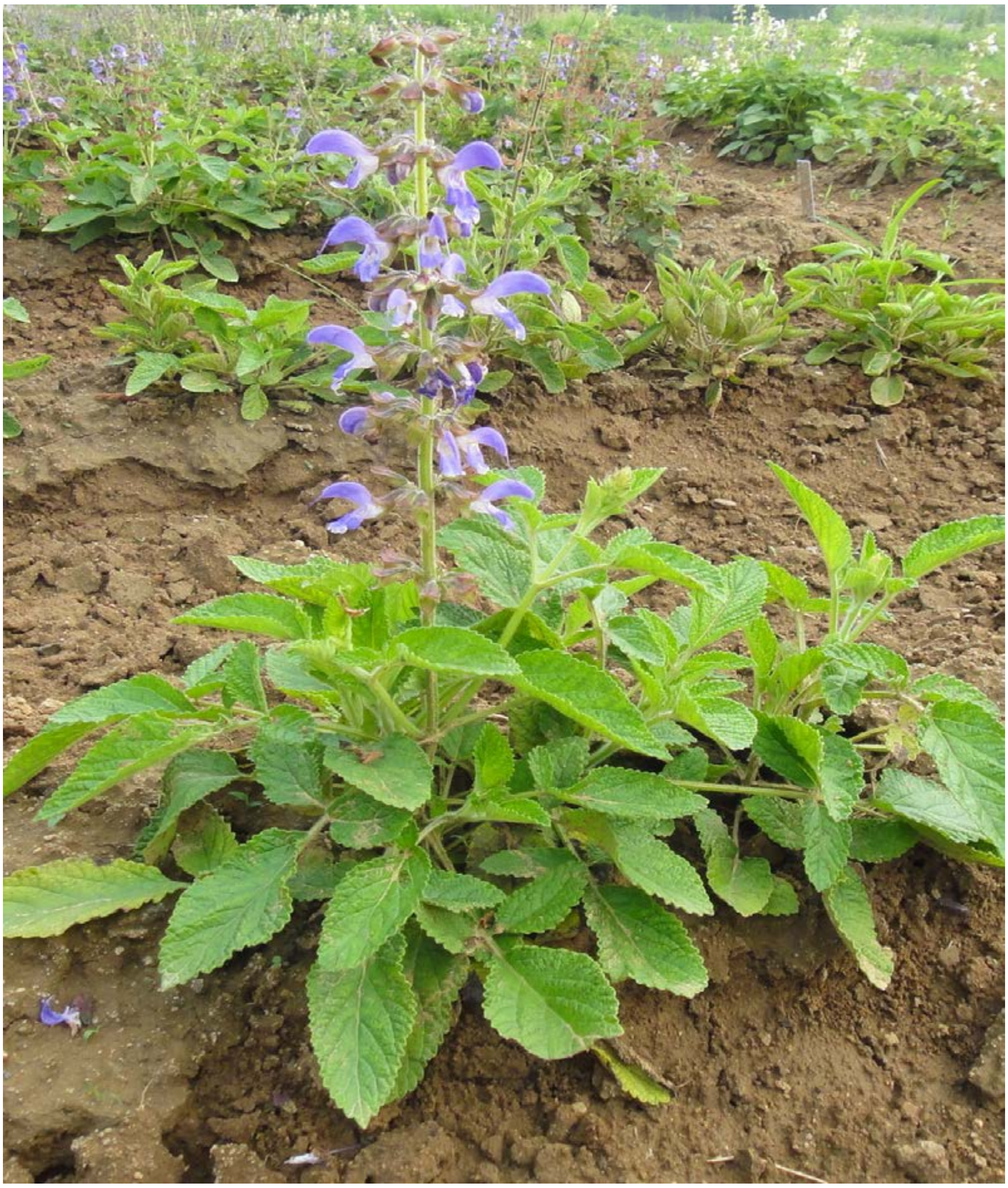

Plant of ZH74

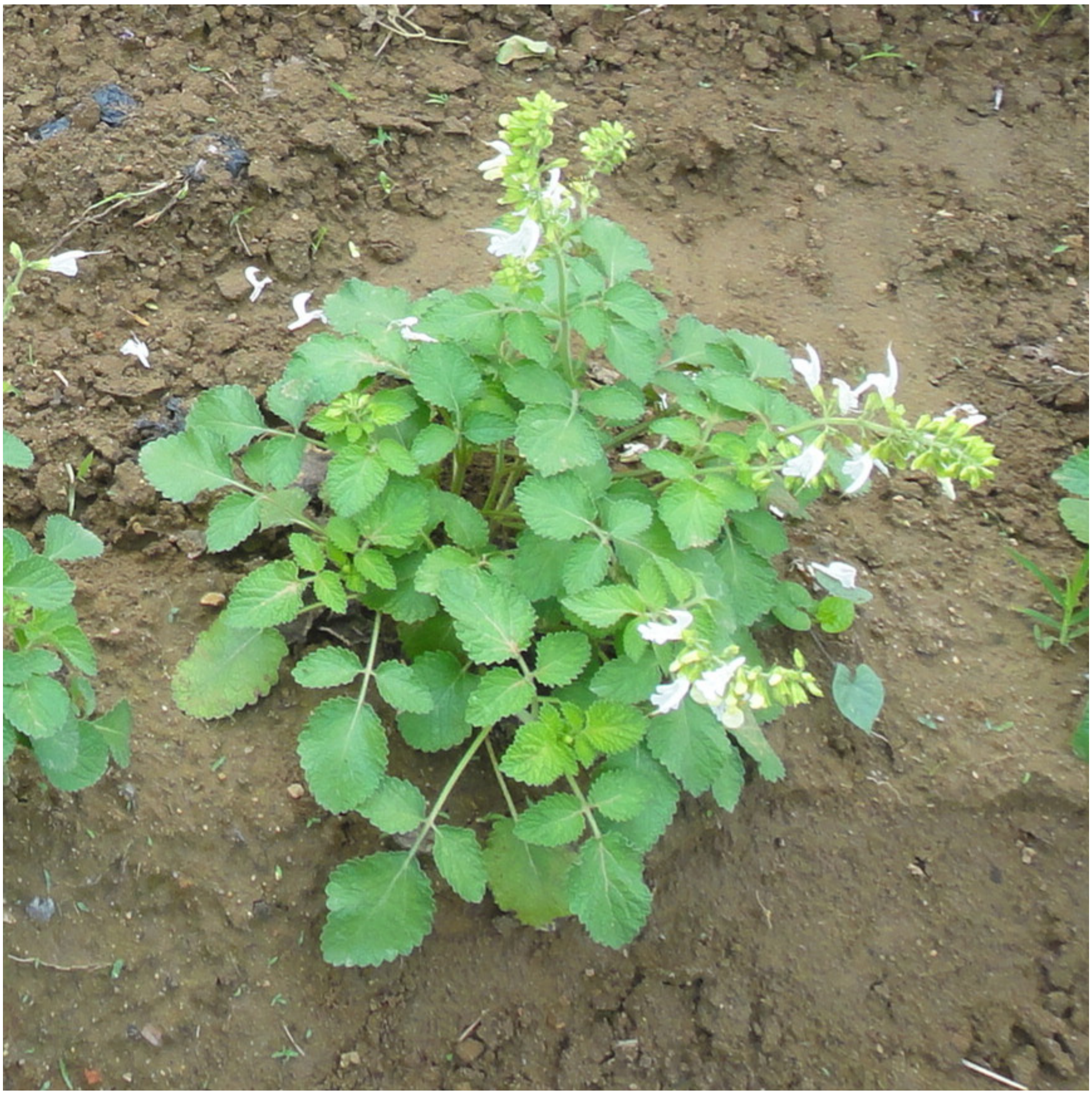

Plant of BH18
